# Significant Decline of Malaria Incidence in Southwest of Iran (2001–2014)

**DOI:** 10.1155/2015/523767

**Published:** 2015-11-16

**Authors:** Shokrollah Salmanzadeh, Masoud Foroutan-Rad, Shahram Khademvatan, Sasan Moogahi, Shahla Bigdeli

**Affiliations:** ^1^Health Research Institute, Infectious and Tropical Diseases Research Center, Ahvaz Jundishapur University of Medical Sciences, Ahvaz, Iran; ^2^Department of Medical Parasitology & Student Research Committee, Faculty of Medicine, Ahvaz Jundishapur University of Medical Sciences, Ahvaz, Iran; ^3^Cellular and Molecular Research Center and Department of Medical Parasitology and Mycology, Urmia University of Medical Sciences, P.O. Box 571551441, Urmia, Iran; ^4^CDC Department, Deputy of Health, Ahvaz Jundishapur University of Medical Sciences, Ahvaz, Iran

## Abstract

Iran is considered as one of the malaria endemic countries of the Eastern Mediterranean Region (EMR) and is at risk due to neighboring Afghanistan, Pakistan in the east, and Iraq to the west. Therefore the aim of the present investigation is the evaluation of the trend of malaria distribution during the past decade (2001–2014) in Khuzestan province, southwestern Iran. In this retrospective cross-sectional investigation, blood samples were taken from all malaria suspicious cases who were referred to health centers across Khuzestan province. For each positive subject a questionnaire containing demographic information was filled out. Data analysis was performed using SPSS 18. From a total of 541 malaria confirmed cases, 498 (92.05%) were male and 43 (7.95%) were female. The highest number of infections was seen in 2001 with 161 (29.75%) cases and the lowest was in 2014 with 0 (0%). Also,* Plasmodium vivax* was identified as dominant species in 478 (88.35%) individuals and* P. falciparum* comprised 63 (11.65%). The highest infection rate was observed in non-Iranian populations with number 459 (84.85%) and imported cases 508 (93.90%). Also, the majority of subjects were over 15 years of age, 458 (84.65%). Due to proximity to endemic countries which has made the malaria campaign difficult, more effort is needed to control the infection in order to achieve malaria elimination.

## 1. Introduction

Malaria is one of the most important infectious diseases, especially in tropical and subtropical areas of the world where 3.3 billion individuals in about 106 countries live at risk. Approximately 200–300 million people worldwide become infected annually and totally 0.6–1 million individuals lose their lives, most of them children under 5 years of age and pregnant women. Malaria is often transmitted through the bite of female Anopheles mosquitoes from one person to another. Other transmission routes include blood transfusion, transfer through the placenta (in the acute stage of infection from mother to fetus), and organ transplantation [1].

Despite considerable progress in the past decades, malaria is still considered a major public health concern in some areas. Malaria in certain regions such as sub-Saharan Africa, Thailand, and India is very prevalent and comprises approximately 95% of total malaria cases throughout the world [2]. In the Eastern Mediterranean Region (EMR), on average more than 10 million clinical cases of malaria occur annually, of which nearly 50,000 cases lead to death. 60% of the EMR populations (287 million people) are at risk [3]. EMR countries, based on malaria control program and disease status, are classified into three groups: the first group, countries that have achieved complete cessation of transmission of malaria including Lebanon, Cyprus, Palestine, Jordan, Qatar, Libya, Bahrain, Tunisia, and Kuwait and also countries where transmission is limited to small regions and is under malaria elimination program including Egypt, Morocco, Oman, Syria, and the United Arabic Emirates (UAE); the second group, countries with intermediate endemicity, including Iran and Saudi Arabia; the third group, countries with extreme transmission of malaria such as Afghanistan, Pakistan, Yemen, Somalia, and Sudan [2]. In addition to climate, other factors such as residence, personal and social lifestyle, cultural and economic status of the region, number of entered migrants to the region, and quantity and quality of malaria control programs in the region hamper the outbreak and spread of malaria [4].

In 1945, the first malaria-training course for preliminary operations of antimalarial campaign was initiated. Then in 1947, for the first time DDT was employed in order to control vectors in hyperendemic villages of Varamin city which led to dramatic reduction in malaria transmission. Afterward, antimalarial campaign was followed during 1948–1956 including antimosquito spraying with DDT, antilarval control measures, drug prophylaxis, and treatment. Eventually in 1957, malaria eradication program was started around Iran and continued till now [5]. During recent years, a significant declining trend has been observed in the rate of malaria incidence throughout Iran. Based on the 2009 World Health Organization (WHO) malaria report, Iran is in pre-elimination phase. Cessation of local transmission of malaria in 2025 is the ultimate goal of the malaria elimination program in Iran [6]. Based on conducted studies in Iran,* P. vivax* has been identified as the major species and* P. falciparum* is more limited to the east and southeast of the country [6, 7]. The south and southeast of Iran are considered as malaria endemic zones and the provinces of Sistan-Baluchestan, Hormozgan, and Kerman with Annual Parasite Incidence (API) between 1 and 8 per 1000 population constitute approximately 95% of total cases, 60% of which were allocated to Sistan-Baluchestan province [3].

*An. Sacharovi*,* An. dthali*, and* An. Stephensi* are identified as definite vectors of malaria in Khuzestan province [8]. Khuzestan province is considered as a Free Trade Zone (FTZ) and agricultural center in Iran and a considerable number of workers are continually deployed to this province. This province, due to its special geographical characteristics and situation, is considered as an immigration zone and in some seasons of the year receives several million tourists and pilgrims. Also, due to its proximity to Iraq, traffic from foreign nationals is observed throughout the year. All of these reasons put this province at risk; thus, the aim of present study was to evaluate the epidemiological status of malaria disease between 2001 and 2014 in the southwest of Iran, Khuzestan province.

## 2. Materials and Methods

### 2.1. Study Area

**Figure 1:**
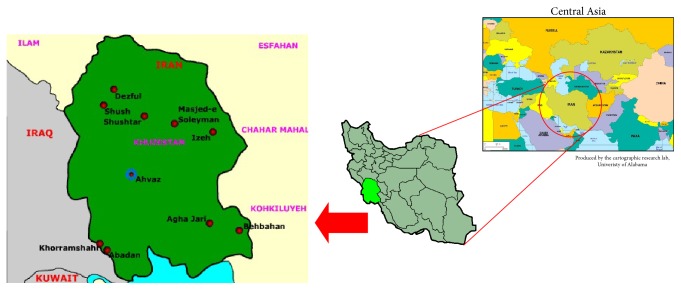
Location of Khuzestan province in Iran (in the southwest of Iran).

This epidemiological study was performed in Khuzestan province, southwestern Iran, located within 29°57′–33°0′N latitude and 47°40′–50°33′E longitude with an area about 64055 km^2^. This province, with a population of 4,531,720 inhabitants (2,286,209 male and 2,245,511 female), is bordered by Iraq in the west, Chahar Mahal and Bakhtiari and Kohgiluyeh and Boyer-Ahmad provinces in the east and northeast, Lorestan province in the north, Bushehr province in the southeast, and the Persian Gulf in the south (Figure 1).

### 2.2. Data Collection

Between 2001 and 2014, from all suspicious malaria subjects who were referred to health centers across Khuzestan province, blood sample was taken by sterile lancet. Afterward, one drop of blood was placed on a microscopic slide in order to prepare peripheral blood smears. Peripheral blood smear after staining by Giemsa was examined by optical microscope in order to detect parasite. A questionnaire including some demographic details such as name, gender, age, residence, nationality (Iranian or non-Iranian), causative agent (*P. vivax*,* P. falciparum*), type of transmission (imported or indigenous), and other details was completed for each positive subject. Eventually all data were gathered from all health centers throughout Khuzestan province and data were analyzed using SPSS software, version 18 (SPSS Inc., Chicago, IL, USA).

## 3. Results

**Figure 2:**
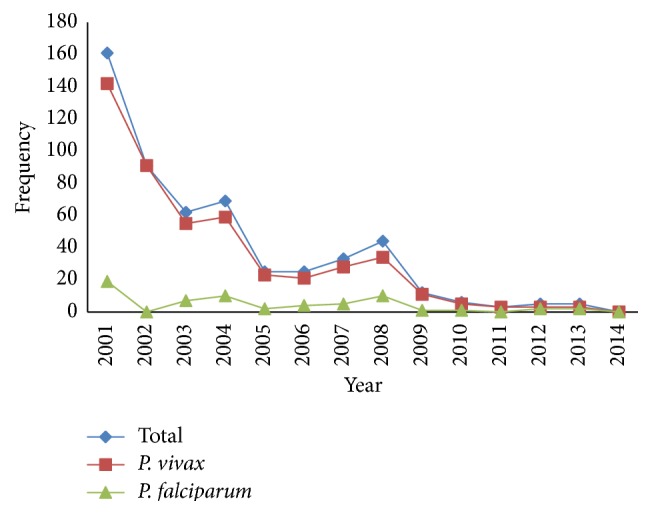
Trend of malaria disease during 2001–2014.

**Figure 3:**
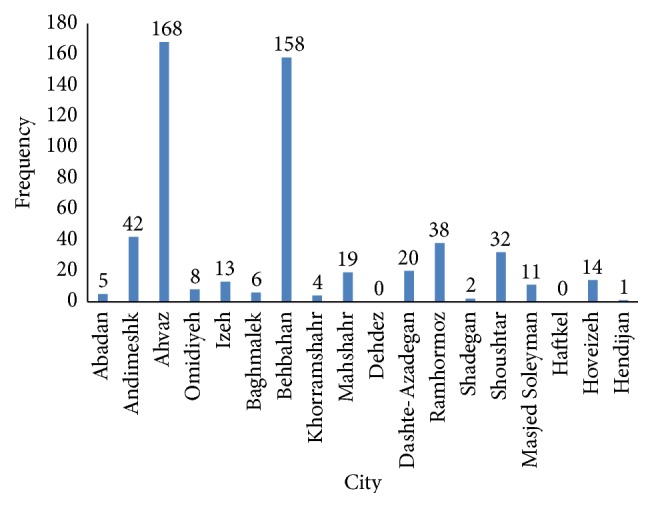
Distribution of malaria infection in different counties of Khuzestan province.

Trend of malaria during the past decade in Khuzestan province has dramatically slumped (Figure 2). The highest number of infections was seen in 2001 with 161 (29.75%) cases and the lowest was in 2014 with 0 (0%). From a total of 541 malaria confirmed cases during 2001–2014, 498 (92.05%) were male and 43 (7.95%) were female.* P. vivax* was the predominant species in 478 (88.35%) individuals and* P. falciparum* species comprised 63 (11.65%). Proportion of infection in different age groups of 0–5, 5–15, and >15 years of age was 26 (4.81%), 57 (10.54%), and 458 (84.65%), respectively. Rate of malaria disease in Iranian and non-Iranian populations was found to be 82 (15.15%) and 459 (84.85%), respectively. Also, number of imported cases was 508 (93.90%) and the number of indigenous cases was 33 (6.10%) (Table 1). Our finding revealed that Ahvaz city (capital of Khuzestan province) with 168 (31.05%) positive cases was in the first rank, Behbahan county with 158 (29.20%) was second, and Andimeshk with 42 (7.76%) was third. In Dehdez and Haftkel counties, no positive cases were reported during the years 2001–2014 (Figure 3). Highest and lowest API were 0.04709 in 2001 and 0 in 2014, respectively (Table 1).

## 4. Discussion

Malaria in developing countries and tropical regions has been of special importance historically [1]. It was estimated in 1924 that, out of 13 million people who live in Iran, 4-5 million of them became infected with malaria and 30–40% of the total deaths were allocated to this infection. Also, 75% of the total Iranian population lived in endemic and hyperendemic areas. At the same time one-third of the budget of the Ministry of Health (MoH) was spent to buy the quinine drug. In 1924 in Tehran, 20% of patients referred to dispensaries had malaria. Also in 1924, 53.5% of all people who lived in Aras river area were diagnosed with malaria and 41.5% of all deaths in this region are allocated to this infection [5]. In a recent study it has been reported that the annual incidence of malaria cases in Iran declined significantly from 66075 to 3200 between 1995 and 2012 [9]. Epidemiological studies have shown that malaria in Iran, unlike Africa and other countries, is dependent on climatic conditions. This means that the unstable situation with rainfall increase may lead to increase in the incidence of disease in high-risk areas [5, 6].

In this study, the trend of malaria disease and its epidemiological features was surveyed between 2001 and 2014. The findings revealed that the rate of infection as a result of malaria campaign has a continuous decreasing trend, so that the frequency from 161 in 2001 fell down to 0 in the end of 2014 in the southwest of Iran, Khuzestan province. Based on current findings, imported cases and non-Iranian patients were found to be 93.90% and 84.85%, respectively. Because of existence of Free Trade Zone (FTZ), the harbor region, and high financial activities in this province, many merchants, investors, and workers from different regions are deployed to these areas annually. Also, this province is a touristic and pilgrimage zone where several thousands of passengers from Iraq country, located at the west of Iran, commute from Khuzestan province annually and this issue could be a reason for high frequency of infection. Salvadó et al. in Spain reported that the main cause of malaria in subjects is migration of the passengers to endemic countries [10]. In another study conducted by Mascarello et al. in Italy during 1990–1998, the number of malaria cases was shown to have increased by 100%, and the main causes of this increase were commute and immigration from endemic areas to the country [11]. Also, based on Iqbal et al.'s study, the rate of malaria infection among immigrants to Kuwait was observed to be 23% [12]. Population movements in malaria endemic zones and the lack of adequate intervention lead to increasing the risk of malaria. For instance, Iran in recent years has hosted approximately 2 million Afghan refugees and unfortunately these refugees have created many serious problems in malaria control program in our country. Entering and traffic of Afghan and Pakistani immigrants to Iran were continued even though Afghanistan and Pakistan are considered as highly dangerous transferors of malaria [6]. Some reports have documented this issue such as Soleimanifard et al. [13] in Isfahan province (91% and 5.6% of total positive cases belonged to Afghan immigrants and other nationalities, resp.), Saghafipour et al. [14] in Qom province (91.5%), Khalili et al. [15] in Yazd province (77.3% in Afghan refugees), and Zia-Sheikholeslami and Rezaeian [16] in Rafsanjan county (98.9% in Afghan refugees).

Current investigation indicated that the number of malaria patients in Khuzestan province, southwest Iran, during the past years has dropped significantly and API in this province ranged from 0.04709 in 2001 to 0 in 2014 which reflects the success in achieving and reaching the malaria control program in this province. Our finding was similar to results gained from Sarafraz et al. [17] in East Azerbaijan province and Ghaffari et al. [18] in Mazandaran province. In the present investigation the rate of infection in males was 92.05% which is consistent with other surveys conducted in Isfahan province [13] (93.5%), Mazandaran province [19] (88.4%), and East Azerbaijan province [17] (86.46%). Also, in some surveys the ratio of both genders was equal and no significant differences were seen between them, such as Alemu et al. [20] in Ethiopia (52.6% male and 47.4% female) and Barak et al. [21] in Ardabil province during 2001–2010 (45% male and 55% female). However, gender is not involved directly and naturally in sensitivity and resistance to malaria but may be correlated with job, type of coating, and cultural habits. Also, social activities and attendance of men in the workplaces outdoors make them more susceptible to mosquito bites and becoming infected.

In the present investigation it was demonstrated that* P. vivax* is the most common species (88.35%) among malaria subjects, which is in agreement with general pattern of disease in Iran [13, 14, 17, 22]. According to the fact that* P. vivax* could relapse due to presence of liver hypnozoites, these patients should be identified and treated. In our study, the highest number was observed in persons over 15 years of age (84.65%), which is in agreement with the results of studies like Saghafipour et al. [14] in Qom province (66.2%), Soleimanifard et al. [13] in Isfahan province, and Bafghi et al. [23] in Yazd province. Presence of infected individuals as disease reservoirs and Anopheles mosquitoes as carriers has an important role in epidemiological status and spread of disease in endemic regions; thus, existence of these factors will help in the transmission of malaria and lead to unstable situation. It should be noted that, despite the significant reduction of malaria cases in Khuzestan province, the hot and humid climate of the province, presence of the Karoun, Maroun, Karkheh, Kheirabad, and Dez rivers, and the arable land surrounding these rivers provide a favorable environmental condition for anopheline mosquitoes larvae development. Evidence shows that malaria control programs with the participation of people in society are more effective compared with those programs that are only designed and implemented by governments; therefore, awareness, attitudes, and behavior of people who live in endemic and hyperendemic areas can be more effective in planning for the control and prevention of malaria [1, 2, 6, 13].

As rapid treatment of infected patients, either Iranian or foreign subjects, some interventions such as antimosquito spraying and antilarval control measures are being carried out in order to eliminate infection throughout the province and, of course, in Iran. Further surveys like spatial studies in order to determine, manage, and analyze epidemiological features using Geographic Information System (GIS) in Khuzestan province are necessary.

## 5. Conclusions

Number of malaria patients during 2001–2014 was decreased significantly in the southwest of Iran, Khuzestan province. Unfortunately, as it was mentioned before, proximity with endemic countries like Iraq, Afghanistan, and Pakistan has made the malaria control program more difficult. Existence of infected people and vectors in the region contributes to the spread of disease around Khuzestan province. According to the specific climatic status in Khuzestan province, the optimum conditions exist for the growth of the Anopheles mosquitoes; thus, the risk of malaria epidemics and incidence of severe cases should be considered constantly. Accordingly, malaria control programs should be continued until disease elimination.

## Figures and Tables

**Table 1:** Epidemiological factors of *malaria* in Khuzestan province during 2001–2014.

Year	Total	API^*∗*^	Causative agent (*n*)		Gender (*n*)		Age (*n*)			Type of transmission (*n*)		Nationality (*n*)	
			*P*.*v* ^*∗∗*^	*P*.*f* ^*∗∗∗*^	Male	Female	0–5	5–15	>15	Indigenous	Imported	Iranian	Non-Iranian
2001	161	0.04709	142	19	151	10	8	21	132	8	153	18	143
2002	91	0.02617	91	0	83	8	8	2	81	1	90	7	84
2003	62	0.01742	55	7	58	4	2	6	54	3	59	12	50
2004	69	0.01922	59	10	65	4	2	5	62	6	63	8	61
2005	25	0.00678	23	2	24	1	0	3	22	0	25	6	19
2006	25	0.00685	21	4	23	2	1	5	19	0	25	5	20
2007	33	0.00903	28	5	30	3	2	3	28	1	32	6	27
2008	44	0.01165	34	10	36	8	1	6	37	8	36	11	33
2009	12	0.00321	11	1	9	3	1	3	8	1	11	2	10
2010	6	0.00155	5	1	6	0	0	0	6	0	6	1	5
2011	3	0.00075	3	0	3	0	1	1	1	0	3	2	1
2012	5	0.00124	3	2	5	0	0	0	5	5	0	3	2
2013	5	0.00124	3	2	5	0	0	2	3	0	5	1	4
2014	0	0	0	0	0	0	0	0	0	0	0	0	0

Total *n* (%)	**541 (100)**		478 (88.35)	63 (11.65)	498 (92.05)	43 (7.95)	26 (4.81)	57 (10.54)	458 (84.65)	33 (6.10)	508 (93.90)	82 (15.15)	459 (84.85)

^*∗*^API: Annual Parasite Incidence = total number of positive slides for parasite in a year × 1000/total population.

^*∗∗*^ *P*.*v*: *Plasmodium vivax*.

^*∗∗∗*^ *P*.*f*: *Plasmodium falciparum*.
